# Adaptation of maize source leaf metabolism to stress related disturbances in carbon, nitrogen and phosphorus balance

**DOI:** 10.1186/1471-2164-14-442

**Published:** 2013-07-03

**Authors:** Urte Schlüter, Christian Colmsee, Uwe Scholz, Andrea Bräutigam, Andreas PM Weber, Nina Zellerhoff, Marcel Bucher, Holger Fahnenstich, Uwe Sonnewald

**Affiliations:** 1 Department of Biology, Division of Biochemistry, Friedrich-Alexander-University Erlangen-Nuremberg, Staudtstr. 5, 91058, Erlangen, Germany; 2 Leibniz Institute of Plant Genetics and Crop Plant Research (IPK), Bioinformatics and Information Technology Group, Corrensstr. 3, 06466, Gatersleben, Germany; 3 Plant Biochemistry Department, Heinrich Heine University Düsseldorf, 40225, Duesseldorf, Germany; 4 Botanical Institute, Albertus Magnus Platz, University of Cologne, 90923, Cologne, Germany; 5 Metanomics GmbH, Tegeler Weg 33, 10589, Berlin, Germany

**Keywords:** Abiotic stress, Nutrient stress, Transcriptome, Metabolome, Ionome, Maize

## Abstract

**Background:**

Abiotic stress causes disturbances in the cellular homeostasis. Re-adjustment of balance in carbon, nitrogen and phosphorus metabolism therefore plays a central role in stress adaptation. However, it is currently unknown which parts of the primary cell metabolism follow common patterns under different stress conditions and which represent specific responses.

**Results:**

To address these questions, changes in transcriptome, metabolome and ionome were analyzed in maize source leaves from plants suffering low temperature, low nitrogen (N) and low phosphorus (P) stress. The selection of maize as study object provided data directly from an important crop species and the so far underexplored C_4_ metabolism. Growth retardation was comparable under all tested stress conditions. The only primary metabolic pathway responding similar to all stresses was nitrate assimilation, which was down-regulated. The largest group of commonly regulated transcripts followed the expression pattern: down under low temperature and low N, but up under low P. Several members of this transcript cluster could be connected to P metabolism and correlated negatively to different phosphate concentration in the leaf tissue. Accumulation of starch under low temperature and low N stress, but decrease in starch levels under low P conditions indicated that only low P treated leaves suffered carbon starvation.

**Conclusions:**

Maize employs very different strategies to manage N and P metabolism under stress. While nitrate assimilation was regulated depending on demand by growth processes, phosphate concentrations changed depending on availability, thus building up reserves under excess conditions. Carbon and energy metabolism of the C_4_ maize leaves were particularly sensitive to P starvation.

## Background

The photoautotrophic life-style of plants depends on availability of light, water and air as well as the balanced access to at least 14 elements in the soil [1]. Limiting supply of one (or more) of these environmental resources leads to abiotic stress. Depending on the nature of the stress, different parts of metabolism are affected, and adaptation to the stress requires readjustment of homeostasis on molecular, cellular and whole plant level [2]. Additionally, protective mechanisms have to be initiated. Independent of the nature of the stress, adaptive processes are realized at the expense of growth. In agriculture, environmental stress is responsible for considerable yield losses every year [3]. Elucidation of the different interacting mechanisms underlying the complex plant stress response is therefore of great general interest [4], and precondition for the improvements of crops and agricultural practices.

Modern transcriptome analysis allows the monitoring of stress related changes on a genomic scale. However, the majority of studies concentrated on one particular stress, and the function of small groups of very stress specific transcripts. This approach makes it difficult to distinguish between specific and general responses. The search for general stress features has been implemented by systematic analysis of transcript data from plants grown under different stress conditions, and identified mainly genes involved in the cross-talk of regulatory systems including transcription factors, Ca^2+^ dependent regulators, protein modifying enzymes, redox regulators, sugar and hormone signaling related features [5,6]. So far, these studies provided only limited information about molecular features directly involved in the down-stream re-adjustment of metabolic pathways and physiological alterations of plants exposed to stress. Detailed investigation of individual cellular processes and the involvement of these processes in the specific or general response could considerably increase our knowledge about molecular changes (transcriptional and other) observed under stress. The parallel analysis of microarrays from different environmental conditions supplemented with metabolite datasets can substantially facilitate data interpretation in this direction. The power of this approach had for instance been demonstrated by Hirai and coworkers [7,8] who characterized a cluster of genes specific for the glucosinolate metabolism, and identified new regulators of the pathways by co-expression analysis.

Plant growth and biomass production are largely connected to activity of the primary metabolism in the source leaf, because it represents the site of carbon (C) fixation in photosynthesis. High plant growth rates also require incorporation of large amounts of nitrogen (N) into amino acids and proteins; and phosphorus (P) for the synthesis of RNA and realization of energy metabolism [9,10]. The plant’s performance under environmental stress is therefore connected to its capacity for re-adjustment of the cellular C-N-P homeostasis.

The aim of this paper is the mechanistic dissection of the metabolic C-N-P balancing system under extreme environmental conditions. For this, maize seedlings were grown under conditions with contrasting effects on N and P metabolism. Deficiencies of N or P would not only affect compounds containing the elements, but would also require re-balancing of metabolism of all other elements which are then available in relatively large amounts. Comparable studies on N and P deficiency stress so far mainly focused on adjustment of C-metabolism. Despite differences in the way the metabolism was perturbed, both nutrient stresses were connected to accumulation of carbohydrates, alteration in source sink allocation and decline in photosynthesis [11-13]. The nutrient stress experiments will be further compared to plants under low temperature stress. This type of stress is not primarily connected to limitation of substrate for synthesis of specific metabolites, but characterized by disturbances in enzymatic kinetics and membrane features [14]. Effects of low temperature stress on some C related pathways are, however, similar to the described effects of nutrients stress, and include accumulation of carbohydrates, changes in carbohydrate utilization and decline in photosynthesis [15-17].

In contrast to model species, maize represents an important crop plant which is used as food, feed and energy source. The fast growth rate and large size of maize plants allow sampling of large amounts of specific tissues. In this study, we concentrate on the metabolism in the source leaf of the plants since it represents the site of synthesis of primary compounds utilized for biomass production. The C_4_ shuttle operating in maize possesses a number of significant consequences for its primary metabolism and tolerance to stress. Carbon dioxide is initially bound to phospho*enol*pyruvate in the mesophyll cells and transported to the bundle sheath, thus allowing enhanced concentrations of CO_2_ at the site of Rubisco and favoring the carboxylase activity of the enzyme. This setup increases the tolerance of the plant to water and N limitation by enhancement of photosynthetic performance under low stomatal conductance and the increased efficiency of C assimilated per amount of protein respectively [18,19]. On the other hand, activity of the C_4_ shuttle depends on enhanced exchange of metabolites between the different cells and compartments [20], and this would increase the complexity of processes involved in maintenance of metabolic homeostasis under stress. The C_4_ enzymes are also more chilling sensitive and performance of maize in low temperatures is impaired [21].

Transcriptional responses to N deficiency, [22-26]; P starvation [13,27-30] or low temperature treatment [16,31-33] have been investigated in a variety of plants species. However, the comparison of data from different experiments is usually problematic because different plant species, different plant tissues, different developmental stages and different stress applications were used. Even very similar experimental setups often reveal considerable variance in transcriptional responses. Yang and colleagues [24] for instance tested the response of various maize genotypes to N starvation and selected a list of marker genes with N stress specific expression patterns. Some of these marker genes exhibited opposite patterns of mRNA accumulation as observed in another N deprivation experiment with maize plants conducted by Amiour and colleagues [25]. To resolve this issue, detailed metadata on experimental conditions, plant development and harvesting procedure will be necessary for the elucidation of the functional relevance of the observed transcriptional changes.

The presented data on transcriptional changes in maize source leaves to N deficiency, P deficiency and low temperature stress is therefore supplemented with phenotype data (shoot biomass, leaf elongation rate) and physiological parameters (gas exchange, PSII fluorescence). The influence of stress severity on the transcript data was reduced by selection of conditions with comparable effects on plants growth; this way the analysis focuses on the different nature of exposed stress, not to its extend. The datasets are further supplemented with data on metabolite profiles for relation of transcriptional changes to behavior of N and P containing compounds but also metabolites from related pathways. Additionally, ion profiles of the leaves are monitored. Especially the nutrient deficiencies strongly influence the performance of the plant’s root and this could have consequences for the uptake and transport of other ions.

The obtained results will be used for identification of (i) molecular processes commonly regulated under different stress conditions, and (ii) metabolic features differently influenced by the experiments. Processes associated with the first group will be analyzed for their possible connection to general stress adaptation, re-adjustment of photosynthesis and growth retardation. The second groups would be particularly relevant for characterization of the N and P homeostasis control under stress, because the system is challenged into different directions by limitation or abundance of selected element. A possible role of the identified processes in the management of C, N and P balance will be discussed.

## Results

### Growth response of maize seedlings to low night temperature, low N and low P stress

Long-term stress conditions with comparable effects on growth parameters were chosen. Under low temperature treatment, reduction in growth started immediately after the onset of stress and the leaf elongation rate was reduced to 59% already at day 20 after germination (Table 1). In nutrient deficient plants, changes in leaf elongation rate developed more slowly. A comparable effect was observed in low N treated plants at day 30 after germination, at the same time-point leaf elongation rate in low P treated plants was reduced to 69% (Table 1). The shoot biomass of the plants was of course influenced by the time of harvest, but comparison between control and stressed plants resulted in similar reduction of the shoot fresh weight. It amounted to 40% under low temperature or low P, and to 30% under low N conditions (Table 1). Controls from the N and P experiment were treated exactly the same, the slight differences in elongation rate represented natural variation between experiments. Maize seedlings from the low temperature experiment were cultivated under slightly different conditions (e.g. different light sources). It had been shown in previous experiments, that growth stage and leaf series had significant influence on the transcriptome and metabolome profiles of maize seedlings, however treatment specific changes differed thereby primarily in the amplitude of the change and not in the direction of the change [26]. For the study of the different nature of stresses we therefore prioritized on experimental setups representing similar effects of growth parameter, and focused data analysis on the comparison of datasets from stressed plants vs. the associated controls.

**Table 1:** Phenotypic characterization of maize seedlings

		**Low temperature**			**Low nitrogen**			**Low phosphorus**		
**Growth feature**	**Unit**	**Control T**	**Cold**	**%**	**Control N**	**Low N**	**%**	**Control P**	**Low P**	**%**
**shoot FW**	g FW	16.23	6.40	**39.42**	60.00	17.58	**29.29**	60.60	24.81	**40.94**
		(±4.25)	(±0.79)		(±3.69)	(±2.18)		(±4.18)	(±2.48)	
**leaf elongation rate**	cm d^-1^	6.51	3.87	**59.38**	7.17	4.35	**60.70**	6.03	4.18	**69.38**
		(±0.65)	(±0.72)		(±0.58)	(±0.61)		(±0.29)	(±0.21)	

Stressed and control plants were always grown within the same experiment. Plants from the low temperature experiment were harvested 20 days after germination; in the nutrient deficiency experiment, plants were harvested 30 days after germination.

### Effects of low temperature, low N and low P stress on photosynthetic gas exchange

Photosynthetic C assimilation provides the basic module for all structural compounds of plant biomass, and measurements of photosynthetic parameters present important information related to general plant performance. Low night temperatures had no negative influence on the measured photosynthetic parameters indicating that the photosynthetic apparatus was intact and C assimilation capacity was not influenced by the treatment (Table 2). Damage to the photosystems as it often occurs when suboptimal temperatures are applied during the photoperiod [34] was not detected in our experiment. When plants were subjected to either N or P deficiency, C assimilation rate and effective PSII quantum efficiency decreased. In low N grown leaves, C assimilation was reduced to 67% and effective PSII quantum efficiency to 86% respectively, indicating reduction in photosynthetic capacity. Stomatal conductance also decreased in low N treated leaves, and this seemed to be a reaction to the reduced C assimilation rate, because the internal CO_2_ concentration in the leaf (Ci) was comparable to control conditions (Table 2). Cultivation of plants under low P conditions, caused a much stronger reduction of photosynthetic capacity of the leaves, and C assimilation as well as effective PSII fluorescence were reduced to 50% or 45% respectively (Table 2). In contrast to the situation under low N stress, stomatal conductance remained on the same level as in the controls causing an increase in the calculated Ci values (Table 2).

**Table 2:** Photosynthetic parameter of maize seedlings

		**Low temperature**			**Low nitrogen**			**Low phosphorus**		
**PS parameter**	**Unit**	**Control T**	**Cold**	**%**	**Control N**	**Low N**	**%**	**Control P**	**Low P**	**%**
**assimilation**	μmol m^-2^ s^-1^	8.00	8.92	**111.51**	8.11	5.46	**67.33**	8.11	4.06	**49.98**
		(±1.29)	(±1.20)		(±1.07)	(±1.41)		(±1.07)	(±1.39)	
**conductance**	mmol m^-2^ s^-1^	74.71	90.25	**120.79**	47.19	32.58	**69.04**	47.19	47.19	**99.99**
		(±10.11)	(±16.54)		(±7.68)	(±7.74)		(±7.68)	(±6.49)	
**Ci**	ppm	161.59	167.24	**103.49**	154.57	166.47	**107.70**	154.57	283.11	**183.16**
		(±12.00)	(±43.79)		(±19.14)	(±17.40)		(±19.14)	(±22.75)	
**PSII efficiency**		0.187	0.224	**119.75**	0.312	0.269	**86.32**	0.312	0.139	**44.56**
		(±0.028)	(±0.046)		(±0.028)	(±0.014)		(±0.028)	(±0.030)	

Gas exchange and fluorescence measurements were performed on leaf 4 at day 20 after germination in the cold stress experiment, and on leaf 6 at days 28–30 after germination in the nutrient experiments. Photosynthesis measurements for nutrient deficient leaves were performed on plants which were cultivated in the greenhouse at the same time and the data for control N and P was therefore the same.

### Ion balance of low temperature, low N and low P stressed leaves

Application of low temperature, low N or low P stress caused significant changes in leaf concentrations of the majority of essential elements (Table 3; Additional file 1: Table S1). Major and minor elements thereby tended to react in the same direction. Of the major elements, S, Mg and Ca were generally reduced in stressed leaves, while K levels increased under the same conditions. A more distinct pattern was observed for N and P. As expected, total N and P concentrations decreased when the relevant ions were deficient in the nutrient solution. However, total N concentrations changed only slightly under low temperature or low P stress. P concentration on the other hand accumulated significantly when plants were subjected to low temperature or low N stress. Mn and Zn showed opposite behavior to P. Concentration of all other minor elements declined under stress. The strongest decrease was observed for Mo, especially under low N stress. In contrast to the minor elements, most measured trace elements accumulated under stress.

**Table 3:** Changes in ion profile under different stress conditions

**element**		**Low T**	**Low N**	**Low P**
major	**N**	−0.199*	−0.993*	0.1431
	**P**	0.858*	1.463*	−1.194*
	**S**	−0.297*	−0.846*	−0.482*
	**K**	0.265*	0.406*	0.197*
	**Mg**	−0.886*	−0.674*	−0.288
	**Ca**	−1.144*	−1.095*	−0.247*
minor	**Mn**	−0.632*	0.003	0.510*
	**Fe**	−0.166	−0.663*	−0.143
	**Zn**	−0.284	−0.719*	0.288*
	**Cu**	−0.289*	−0.813*	−0.100
	**Mo**	−1.702*	−3.486*	−0.509*
	**Sr**	−1.306*	−1.140*	−0.241*
trace	**Li**	nd	−1.804*	−0.214*
	**V**	0.667	0.620	0.253*
	**Cr**	3.044	0.463	0.403
	**Co**	nd	0.542	−0.203
	**Ni**	0.757	0.882*	0.751*
	**As**	−1.198*	−0.466	−0.366*
	**Rb**	0.375	0.363*	0.406*
	**Cs**	−0.290	0.626*	0.114
	**Pb**	−0.675	−1.488*	nd
	**Cd**	−0.606*	−2.050*	0.229

Data is shown as log_2_ fold change of stressed vs control values. The asteriks mark significant changes (*t*-test, p < 0.05); nd indicates that the measurement was below detection limit (not determined); *low T* low temperature, *low N* low nitrogen, *low P* low phosphorus.

### Metabolite profiles of low temperature, low N and low P stressed leaves

**Figure 1:**
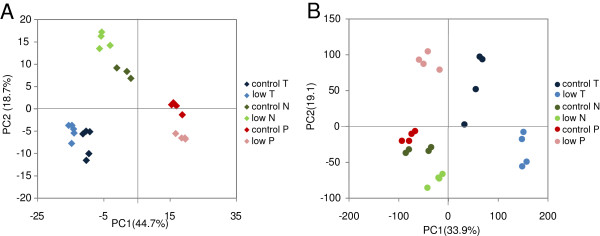
**Principal component analysis.** PCA was performed using **(A)** 539 metabolites peaks (153 known plus 386 unknown) and **(B)** 31501 transcript features.

The general influence of low temperature, low N and low P stress on the metabolite profile (known and unknown metabolites) of maize source leaves was first tested by PCA. Figure 1A shows that experiment specific variation lay behind the first principle component (PC1 representing 44.7% variation). Experimental differences in metabolite profiles had been observed regularly and can be explained by the influence of small environmental differences [35]. In our work, differences in soil batches, seed batches or the influence of age on the performance of lamps in the growth chambers could have contributed to the experimental differences. Plants from the low temperature experiment were harvested at an earlier growth stage and were grown in different chambers. These aspects were also expected to have influence on separation of samples along PC1. Within each experiment there was a clear separation of samples from control and stressed plants. Differences between low temperature stressed and associated control T samples were also found along the PC1 axis, while samples from the nutrient experiments on the other hand were separated along PC2 (18.7%). The control samples from both nutrient experiments clustered together in the middle of PC2, and low N and low P samples were placed on opposite sides of the controls indicating that N and P deficiencies had contrasting effects on metabolite pattern.

**Figure 2:**
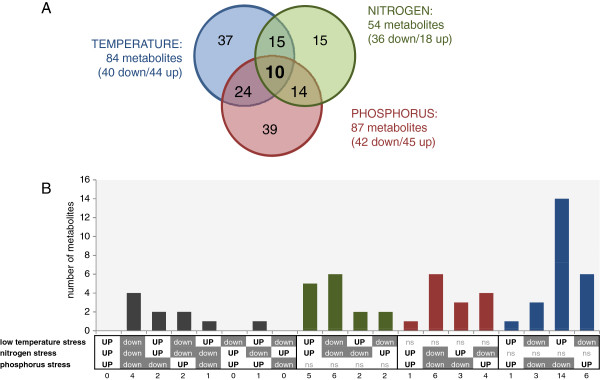
**Intersection of significantly regulated known metabolites under different stress conditions. (A)** Venn diagram of intersection between metabolites regulated by low temperature, low N and low P stress, **(B)** Overview of direction of regulation of metabolites responding to at least two stress situations (ns = not significant).

**Figure 3:**
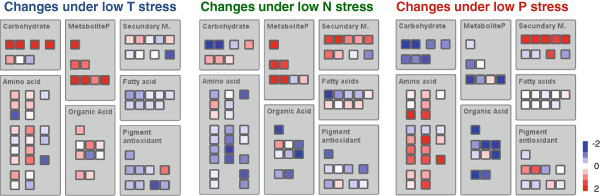
**Changes in metabolites from different biochemical groups under low temperature, low N and low P stress.** Data is presented as heatmaps for log2 fold changes in stressed vs control conditions. The squares indicate the following metabolites: carbohydrate (1st line: glucose, fructose, sucrose, starch; 2nd line: raffinose, melibiose, stachyose); amino acid (1st line: glutamate, glutamine, alanine; 2nd line: aspartate, asparagine; 3rd line: glycine, serine; 4th line: arginine, proline, cysteine; 5th line: threonine, isoleucine, lysine; 6th line: valine, leucine; 7th line: tyrosine, phenylalanine, tryptophane; 8th line: oxoproline, GABA; 9th line: ornithine, beta-alanine); MetaboliteP (1st line: phosphate; 2nd line: ribose-5-P, sedoheptulose-7-P; 3rd line: glucose-6-P, fructose-6-P, glucose-1-P, UDPglucose); organic acids (1st line: pyruvate; 2nd line: citrate, cis-aconitate, 2- oxoglutarate; 3rd line: succinate, fumarate, malate; 4th line: glycerate, glyoxylate); secundary metabolites (1st line: shikimic acid, quinic acid, chlorogenic acid, neochlorogenic acid, coumaronyquinic acid; 2nd line: trans-caffeic acid, trans-ferulic acid, DIMBOA, salicylic acid); fatty acid (1st line: lauric acids (C12:0), myristic acid (C14:0), palmitic acid (C16:0), stearic acid (C18:0), eicosanoic acid (C20:0); 2nd line: behenic acid (C22:0), lignoceric acid (C24:0), cerotic acid (C26:0), montanic acid (C28:0)); pigment/antioxidant (1st line: chlorophyll; 2nd line: zeaxanthin, antheraxanthin, violaxanthin, ascorbic acid; 3rd line: alpha-tocopherol, gamma-tocopherol, beta-carotin, lutein).

Metabolite peaks with known identity were used for further analysis. Low temperature and low P stress caused significant changes (*t*-test, p < 0.05) in 84 and 87 metabolites respectively. About the same number of metabolites increased and decreased under these stresses (Figure 2). N limitation resulted in significant changes of 54 known metabolites, of these more (36) were reduced than enhanced (18). Figure 3 summarizes the changes in metabolite patterns of the most important metabolite groups in source leaf metabolism. N containing amino acids and P containing phosphorylated metabolites are reduced when the specific element was limiting. Phosphorylated metabolites generally reacted in the same direction as the soluble Pi concentration (Figure 3, Additional file 2: Figure S1). The overview also showed that most metabolites from a biochemical group usually reacted in the same direction.

Low temperature stress generally resulted in increases in pools of carbohydrates such as sugars (glucose, fructose, sucrose, raffinose) and starch, but also phosphorylated metabolites and organic acids accumulated. Concentrations of most amino acids were also slightly enhanced (Figure 3). Proline, which represents a stress marker for different abiotic stress situations, showed a particularly pronounced increase (Additional file 3: Table S2). General decreases could be observed for all shown fatty acids and pigments. The majority of measured secondary compounds were not influenced by low temperature treatment.

Besides the decrease in amino acids, N limitation also caused reductions in the pools of organic acids (with exception of citrate), medium to long chain fatty acids as well as pigments (Figure 3). Phosphorylated metabolites and all phenylpropanoid secondary metabolites increased under low N stress. The picture for the most important carbohydrates of primary leaf metabolism was more complex under low N conditions (Figure 3). While the sugars glucose, fructose and sucrose decreased, sugars from the raffinose family accumulated, starch concentration also increased in leaves under low N conditions.

Under low P treatment, the pattern of primary metabolites looked almost opposite to the picture obtained under low temperature. Carbohydrates, phosphorylated metabolites and organic acids, which had increased under low temperature stress, were reduced by low P stress (Figure 3; Additional file 2: Figure S1). Some protective pigments such as zea- and antheraxanthin, which had decreased under low temperature conditions, were enhanced under low P. Only amino acids responded in the same direction under both stress treatments, but the degree of change was more pronounced under low P, especially high was the increase of glycine and serine (Additional file 3: Table S2). Phenylpropanoid related secondary metabolites also accumulated under low P stress similar to the situation caused by N limitation. Fatty acids were not influenced by the low P stress.

The Venn diagram (Figure 2A) revealed that only ten metabolites were actually significantly altered in all three applied stress conditions. Only four of these changed in the same direction and were commonly reduced, namely phytylesters (including chlorophyll a and b), salicylic acid, digalactosylglycerol and galloylhexose (Figure 2; Table 4). No metabolite marker with positive correlation to the applied stresses could be identified. All other metabolites from the intersection reacted differently to the applied stresses. Glucose-6P, a metabolite with central role in primary carbohydrate metabolism, was accumulated strongly under low temperature and low N stress, but decreased under low P. As described above, most other measured phosphorylated metabolites and to some degree also starch followed the same pattern as glucose-6P (Figure 3). Another significantly changed metabolite with very central function in metabolism was the organic acid pyruvate. Its pool was enhanced under low temperature stress and reduced under low P and low N conditions. The majority of other measured organic acids followed the same response pattern (Figure 3). Phenylalanine increased under low temperatures and low P stress, but decreased under low N stress thus representing the general response pattern of most amino acids. The carotenoid lutein serves in the chloroplast as photoprotective compound, in the present study it was only increased by low P stress, and decreased under low temperature and low N. Intersections between only two stress situations revealed overlap for regulation of 24, 15 and 14 metabolites under low temperature and low P, low temperature and low N, and low N and low P respectively (Figure 2A).

**Table 4:** Metabolites significantly changed by low temperature, low N and low P stress

**Metabolite name**	**Biochemical group**	**Low T**	**Low N**	**Low P**
Phytylesters (additional: Chl a, Chl b)	Pigments and antioxidants	−0.692	−1.156	−0.277
Salicylic acid	Phytohormones	−1.254	−0.872	−0.711
Digalactosylglycerol	Lipids, fatty acids	−0.654	−0.931	−0.922
Galloylhexose	Secondary metabolism	−1.895	−0.886	−0.984
7-OH-HBOA-2-O-glucoside	Secondary metabolism	−0.738	−1.134	1.311
Lutein	Pigments and antioxidants	−0.454	−0.368	0.41
Pyruvate	Organic acids	0.641	−1.426	−2.482
Glucose-6-phosphate	Carbohydrates	3.086	2.36	−1.487
Serine, lipid fraction	Lipids, fatty acids	0.576	0.766	−1.416
Phenylalanine	Amino acids	1.109	−1.22	1.438

The values represent log_2_ data of fold changes between stressed and control samples.

### Transcript profiles of low temperature, low N and low P stressed leaves

Differences in transcript patterns of low temperature, low N, and low P stressed leaf samples and the associated controls were also tested by PCA. The first principal component (33.9%) separated samples from the low temperature experiment from the nutrient experiment samples (Figure 1B). Since plants from N and P experiments were harvested at a later growth stage and were grown in different chambers than in the low temperature experiment, this component could be influenced by developmental differences. The second principal component (19.1%) separated low N and low P samples from the controls from both experiments. Experimental differences were still visible but much less pronounced than in the analysis of metabolite profiles. Again, low P and low N treated samples clustered on opposite sides of the controls.

**Figure 4:**
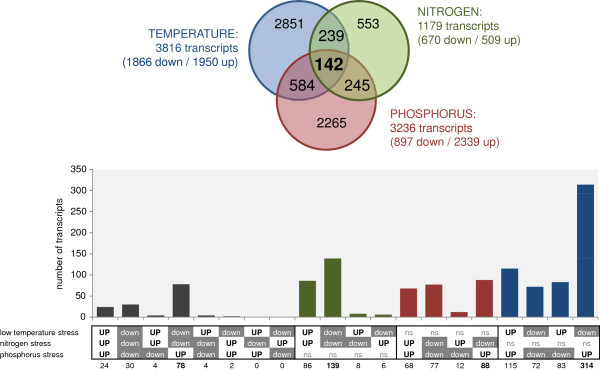
**Intersection of significantly regulated transcripts under different stress conditions. (A)** Venn diagram of intersection between transcripts regulated by low temperature, low N and low P stress, **(B)** Overview of direction of regulation of transcripts regulated in at least two stress situations (ns = not significant).

Overall, 3816, 3236, and 1179 transcripts were significantly changed by low temperature stress, P stress and N stress respectively (Figure 4, Additional file 4: Table S3). About equal amounts of transcripts were down- and up-regulated by low temperatures. N deficiency caused more down- than up-regulation of transcripts. The opposite was true for P deficient sample and about 2.5 times more transcripts were up- than down-regulated (Figure 4). The GO-term enrichments tool (AgriGO; [36]) indicated that low temperature caused reduction in transcripts related to transport processes, protein modification, lipid and carbohydrate metabolism (Table 5). Nitrogen, protein, polysaccharide metabolism and ribosomal protein related transcripts on the other hand were up-regulated (Table 5; Additional file 5: Table S4). Gene expression related transcripts such as transcription factors were affected in both directions by low temperature stress (Table 5). N deficiency was characterized by down-regulation of transcripts involved in phosphate transport, oligosaccharide biosynthesis and amine metabolism (Table 5). In contrast, under low P stress functional enrichment was only found for the up-regulated features, among them transcripts from oxidative stress, polysaccharide metabolism, phosphate transport, cell wall degradation and alcohol metabolism (Table 5).

**Table 5:** GO term enrichment in transcript groups significantly regulated by low temperature, low N or low P stress

**Stress**	**Regulation**	**Process**	**Function**	**Compartment**
LOW T	down	anion transport	heme binding	none significant
		phosphate transport	tetrapyrrole binding	
		organic acid transport	phosphoric ester hydrolase	
		amino acid phosphorylation	anion transmembrane transporter	
		protein modification	phosphate transmembrane transporter	
		lipid metabolism	protein kinase activity	
		carbohydrate metabolism	transcription factor regulation	
		gene expression	oxidoreductase regulation	
		regulation of nitrogen metabolism	lipase activity	
			sugar binding	
	up	translation	glycosyl bond hydrogenase	ribosome
		protein metabolism		cytoplasm
		nitrogen compound metabolism		
		gene expression		
		polysaccharide metabolism		
LOW N	down	nitrogen compound metabolism	phosphoric ester hydrolase	none significant
		phosphate transport	phosphate transmembrane transport	
		oligosaccharide biosynthesis	phosphatase	
		trehalose biosynthesis	heme binding	
		amine metabolism	tetrapyrrole binding	
	up	none significant	glycosyl group transferase	none significant
			enzyme regulator activity	
			nucleoside-triosephosphatase regulation	
LOW P	down	none significant	none significant	none significant
	up	response to oxidative stress	oxidoreductase	cell wall
		lipid metabolism	heme binding	
		polysaccharide metabolism	tetrapyrrole binding	
		nitrogen compound metabolism	antioxidant activity	
		phosphate transport	lipase activity	
		cell wall degradation	phosphoric ester hydrolase	
		alcohol metabolism	phosphate transmembrane transport	
			phosphatase activity	
			chitinase activity	

Analysis was performed using the AgriGO webtool [36]. The results were summarized to avoid repetition.

A total of 142 transcripts were significantly changed under all three applied stress conditions. Of those 30 and 24 were commonly down- and up-regulated respectively (Figure 4; Table 6, Additional file 6: Table S5). Among the commonly down-regulated genes only two nitrate reductases and an amino acid transporter would translate into proteins with direct metabolic function. Most other commonly down-regulated transcripts are not very well described for maize, but are predicted to have regulatory functions such as kinases or transcription factors. Transcripts with predicted regulatory function also dominate the general positive stress markers (Table 6). The MYB transcription factor rough sheath *RS2* (GRMZM2G403620) has already been described as a negative regulator of maize leaf development [37]. Two additional stress stimulated transcripts were predicted to be involved in structural assembly of chloroplasts (chaperonin: AC215201.3_FG005; GSA2: GRMZM2G116258).

**Table 6:** Transcripts significantly changed into the same direction by low temperature, low N and low P stress

			**LOG**_**2 **_**FC stress vs control**		
**OptiID**	**Parent 4a.53**	**Short description**	**Low T**	**Low N**	**Low P**
OptiV1C13059	GRMZM2G038281	Glycosyl hydrolases family 35	−1.375	−1.633	−1.779
OptiV1C01796	GRMZM2G430936	Glycosyl hydrolases family 18	−2.416	−1.964	−1.302
OptiV1S19512	GRMZM2G052625	Glutathione S-transferase	−3.028	−2.607	−1.172
OptiV1S33411	GRMZM2G050307	SAM dependent carboxyl methyltransferase	−3.403	−1.785	−1.378
OptiV1S28200	GRMZM2G133996	SAM dependent carboxyl methyltransferase	−3.481	−1.774	−1.294
OptiV1S33603	GRMZM2G050321	SAM dependent carboxyl methyltransferase	−3.407	−1.725	−1.303
OptiV1C06941	GRMZM2G048904	GDSL-like Lipase/Acylhydrolase superfamily	−1.238	−1.545	−2.086
OptiV1C08466	GRMZM2G142386	cytosolic isoform of nitrate reductase	−1.203	−3.109	−1.166
OptiV1S19330	GRMZM2G076723	nitrate reductase	−1.190	−3.012	−1.201
OptiV1C15441	GRMZM2G110195	transmembrane amino acid transporter	−1.349	−1.785	−1.147
OptiV1S27277	GRMZM2G332660	calcium-dependent protein kinase	−2.708	−1.947	−2.072
OptiV1S18644	GRMZM2G075286	wall-associated kinase	−3.521	−5.376	−3.073
OptiV1S18504	GRMZM2G173710	histidine-containing phosphotransmitter	−3.184	−1.447	−2.076
OptiV1S27555	GRMZM2G401606	S-locus lectin protein kinase	−4.052	−2.573	−1.641
OptiV1C01433	GRMZM2G086066	Heavy metal transport/detoxification superfamily	−2.426	−2.280	−1.938
OptiV1S26323	GRMZM2G010920	myb-like HTH transcriptional regulator	−1.174	−2.835	−1.170
OptiV1C00704	GRMZM2G086231	2OG-Fe(II) oxygenase superfamily	−3.268	−4.826	−3.697
OptiV1C04996	GRMZM2G168552	abscisic stress-ripening, putative	−1.088	−1.102	−1.313
OptiV1C11463	GRMZM2G122954	endosomal targeting BRO1-like domain-containing protein	−1.399	−2.072	−1.815
OptiV1C02712	GRMZM2G166906	Glucose-methanol-choline (GMC) oxidoreductase	−4.346	−4.293	−1.252
OptiV1S19822	GRMZM2G079616	HXXXD-type acyl-transferase family protein	−2.505	−3.119	−1.238
OptiV1S26828	GRMZM2G057467	Cytochrome b561/ferric reductasetransmembrane domain	−2.315	−6.245	−3.414
OptiV1S34472	GRMZM2G428119	BTB-POZ and MATH domain 4	−2.438	−1.392	−1.525
OptiV1S24670	GRMZM2G073969	unknown	−4.009	−3.256	−1.747
OptiV1S19308	GRMZM2G134219	unknown	−1.834	−3.057	−1.950
OptiV1C14939	GRMZM2G470882	unknown	−3.374	−2.211	−1.400
OptiV1S18877	GRMZM2G439246	unknown	−1.011	−1.636	−1.279
OptiV1C11114	GRMZM2G111324	Glucan endo-1,3-beta-D-glucosidase	1.738	1.529	1.098
OptiV1S18343	GRMZM2G090441	Glycosyl hydrolases family 18	5.732	2.414	1.432
OptiV1C04728	GRMZM2G062974	basic chitinase	1.656	2.002	2.282
OptiV1S31825	GRMZM2G046750	Bifunctional inhibitor/lipid-transfer protein	5.542	3.523	2.572
OptiV1C01291	GRMZM2G396212	Class-II DAHP synthetase family	1.621	1.162	1.804
OptiV1S28228	AC215201.3_FG005	chaperonin-60 alpha involved in Rubisco folding	1.847	1.664	1.363
OptiV1S23488	GRMZM2G116258	glutamate-1-semialdehyde 2,1-aminomutase 2 (GSA2)	1.174	1.057	1.158
OptiV1S33616	GRMZM2G072569	Leucine-rich receptor-like protein kinase	1.061	1.359	1.324
OptiV1C17401	GRMZM2G017355	Mitochondrial transcription termination factor	1.142	1.179	1.208
OptiV1S23917	GRMZM2G171466	Transcription factor containing NAC and TS-N domains	1.146	1.582	1.391
OptiV1C00118	GRMZM2G403620	MYB transcription factor (rough sheath2)	3.127	1.987	1.818
OptiV1C02069	GRMZM2G132169	Multicopper oxidase/laccase	3.789	1.599	1.736
OptiV1C09392	GRMZM2G391042	Calcium transporting ATPase	1.357	1.057	1.201
OptiV1S19459	GRMZM2G120897	Actin filament-coating protein tropomyosin	5.247	1.336	1.461
OptiV1S30839	GRMZM2G150791	Ankyrin repeat	1.128	1.559	1.202
OptiV1C14068	GRMZM2G060029	Armadillo/beta-catenin-like repeats	2.081	2.121	4.472
OptiV1C03999	GRMZM2G154414	Cyclin-dependent kinase inhibitor family	2.470	2.813	1.220
OptiV1S20962	GRMZM2G143457	unknown	1.453	2.321	1.437
OptiV1C15394	GRMZM2G004349	VIRB2-interacting protein 1 (BTI1)	1.640	1.372	1.600
OptiV1C13551	GRMZM2G015603	alpha/beta hydrolase superfamily	3.798	2.658	2.490
OptiV1C10502	GRMZM2G095404	Peroxidase superfamily	5.299	3.578	2.478

The values represent log_2_ data of fold changes between stressed and control samples. Transcripts appearing twice in the list but representing the same genome loci were reduced to one entry, transcripts without localization on the current genome (4a.53) were omitted. The complete list can be accessed in the Additional file 6: Table S5.

The bulk of commonly regulated transcripts (55%) followed the pattern: down-regulated under low temperature and low N, but up-regulated under low P conditions (Figure 4). This pattern was dominated by transcripts which had been previously described for their involvement in regulation of phosphate homeostasis, such as SPX domain proteins, phosphate transporters, phosphoesterases or sulfolipid synthesis related proteins [38]. They amounted to 41% of the functionally annotated transcripts in this group (Additional file 6: Table S5). The majority of transcripts with significant regulation in only two of the applied stress situations actually followed the same pattern: common down-regulation under low N and low temperature, but up-regulation under low P (Figure 4). Generally, 584, 245 and 239 transcripts were significantly regulated by low temperature and low P, low P and low N, or low N and low temperature respectively (Figure 4).

### Effects of low temperature, low N and low P stress on transcripts of primary C, N and P metabolism of source leaves

**Figure 5:**
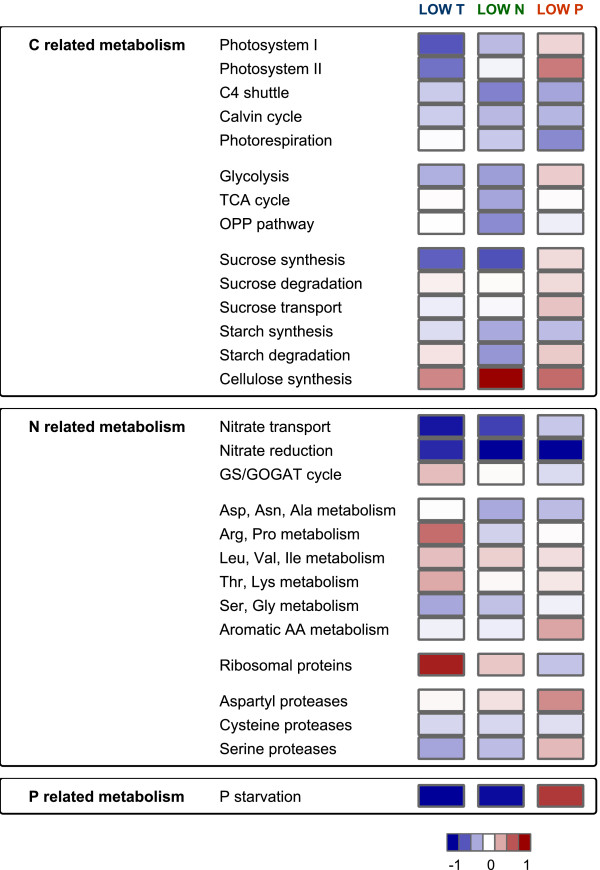
**Summarised transcriptional changes in primary metabolism under low temperature, low nitrogen or low phosphorous stress.** Data is presented as log_2_ values of fold changes (stressed vs control) from the average changes in the pathway. The data behind the average values are presented in Additional file 5: Table S4.

The regulation of primary C, N and P metabolism under stress had been the specific focus of this study. Therefore, transcripts involved in the selected pathways were singled out and associated with the features on the maize microarray chip. The mean change of transcripts from a particular pathway is summarized in Figure 5, and data from the individual sequences can be found in supplements (Additional file 5: Table S4).

The majority of transcripts belonging to both photosystems were not changed significantly under stress, nevertheless the transcripts all tended to react in the same direction indicating high transcriptional coordination. Low temperature and to a less degree also low N stress caused down-regulation of photosystem related transcripts, but low P initiated enhanced transcription. Transcripts involved in the C_4_ specific shuttle tended to be down-regulated under all applied stress conditions, and a similar trend was observed for Calvin-Benson cycle related transcripts. Photorespiration related transcripts were affected strongest in low P treated leaves.

Under N deprivation, a general transcriptional reduction was also characteristic for pathways of C catabolism such as glycolysis, TCA cycle and oxidative pentose phosphate (OPP) pathway. In contrast, individual transcripts from glycolysis showed an upwards trend under low P conditions. Particularly strong reaction to all three stress conditions was observed for two transcripts representing glycolytic bypass reactions, a PPi-dependent phosphofructokinase (GRMZM2G059151) and housekeeping PEPC (GRMZM2G110714). Their abundance decreased under low temperature and low N, but increased under low P (Additional file 5: Table S4).

Sucrose and starch are the main products of C assimilation in the source leaf. Sucrose synthesis and transport seemed to be down-regulated under low temperature and low N. Several sucrose synthesis related transcripts such as UDP-glucose pyrophosphorylase (AC197705.4_FG011) and sucrose phosphate synthase (GRMZM2G013166, GRMZM2G008507, GRMZM2G140107; see Additional file 5: Table S4) were reduced significantly in low temperature and low N conditions, but not affected by low P. The maize sucrose transporter SUT1 (GRMZM2G034302), which is responsible for phloem loading in maize leaves [39] showed a similar expression pattern. Expression of sucrose degradation related transcripts was characterized by more individual responses to the stress conditions. Strong responses were found for few sucrose degrading genes. A vacuolar invertase transcript (GRMZM2G089836) was stimulated by low temperature, and this mirrors accumulation of the reaction products glucose and fructose (Figure 3). The same invertase transcript was reduced under low C status in P deficient leaves. A cell wall invertase transcript on the other hand was induced only by low P (GRMZM2G139300). Two sucrose synthase transcripts (GRMZM2G060659; GRMZM2G045171) were up-regulated by all three stress conditions, and the correlation in their expression patterns to the majority of cellulose synthase transcripts suggests that they were probably both involved in cell wall metabolism [40]. There was no correlation between starch content of leaves and expression of starch metabolism related transcripts. Transcripts of starch synthases tended to be generally reduced in all three stress experiments (Additional file 5: Table S4).

**Figure 6:**
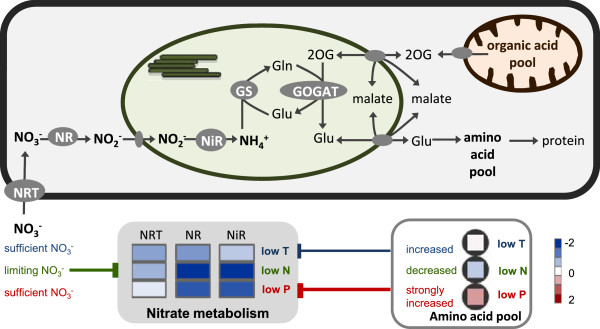
**Regulation of nitrate metabolism under low temperature, low nitrogen and low phosphorus stress.** Transcription of nitrate transporters (NRT), nitrate reductase (NR) and nitrite reductase (NiR) are down-regulated under all three stress conditions, but by different control mechanisms. Under low N the limited availability of nitrate causes down-regulation of nitrate metabolisms, under low temperature and low P conditions on the other hand increases in the amino acid pool were responsible for control of nitrate metabolism. Heat maps show log2 values of fold changes stress/control; data behind NRT and NR are mean values from more than one transcript (see Additional file 5: Table S4); data for amino acid pool averages values from all measured amino acids. [GS- glutamine synthase; GOGAT – glutamate synthetase; 2OG – 2-oxoglutarate].

The reaction of transcripts directly involved in nitrate transport and assimilation was a uniform down-regulation under stress (Figures 5 and 6). Especially under low N and low P stress, all identified sequences for nitrate and nitrite reduction were significantly reduced; under low temperature stress, the reaction was slightly less prominent. Transcripts of the connected ammonium assimilating GS/GOGAT cycle did not follow the same pattern and were mainly unchanged by stress treatment. The chloroplast GS2 even tended to be generally transcriptional up-regulated (Additional file 5: Table S4). There was no uniform picture for amino acid biosynthesis related transcripts in the presented experiments. Interesting was however the behavior of proline synthesis. The final enzyme of proline biosynthesis (P5SC: delta 1-pyrroline-5-carboxylate synthase) was up-regulated under cold stress (Additional file 5: Table S4), and this correlated with significant increase in proline concentration of the leaf (Figure 3). The reaction was very specific for low temperature stress; N or P stress was not characterized by proline accumulation. Under low N, P5SC transcripts even seemed to be down-regulated, and this was connected to a decrease in proline content. P deficiency did not cause any significant changes in proline metabolism (Figure 3). In the source leaf amino acids are further incorporated into proteins, and the coordinated expression of ribosomal proteins usually gives a good indication of the activity of protein biosynthesis. In the presented experiments, ribosomal protein transcripts increased considerably under low temperature stress. The same tendency was also observed under low N stress, but low P caused reduction in abundance of ribosomal protein transcripts (Figure 5, Additional file 5: Table S4). Protein degradation usually increases under stress for remobilization of protein reserves and also represents part of senescence related processes. The expression of main protease groups and other senescence markers was investigated but there was no evidence for a general induction of senescence in the examined source leaves (Additional file 5: Table S4).

**Figure 7:**
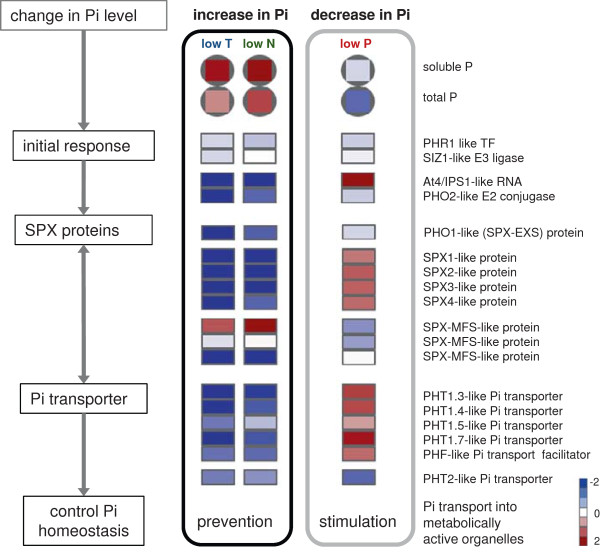
**Regulation of phosphate homeostasis under low temperature, low nitrogen and low phosphorus stress.** Leaf concentrations of soluble P (inorganic and organic P) and total P increased under low temperature and low nitrogen stress, but decreased under low P stress. The response of transcripts for initial P starvation response, SPX proteins and phosphate (Pi) transporters is shown as heat maps presenting log2 values of fold changes stress vs control. Sequences with similar expression patterns and the same predicted homologue in Arabidopsis are summarised as mean. Data for the individual sequences behind the heatmaps are listed in Additional file 5: Table S4.

Remarkably strong was the reaction of many transcripts previously described for their up-regulation under P starvation [38]. The increased expression of the majority of these genes under low P conditions confirms their importance in regulation P deficiency (Figure 7). It had previously been shown [26] that many P starvation genes are significantly down-regulated under N deficiency, when phosphate concentrations increased in the leaves. In this study, phosphate accumulation and regulation of related transcripts was also observed under low temperature stress. Only minor changes were observed for transcription of phosphate balance regulators (PHR1 and SIZ1-like proteins), but the reaction to stress was strong for many P starvation response genes (Figure 7, Additional file 5: Table S4). Only one SPX-MFS domain protein had an expression pattern opposite to the majority of response genes. It was reduced by low P and stimulated under high P status under low temperature and low N conditions.

## Discussion

### General impact of low temperature, low N and low P stress on metabolite and transcript profiles

Examination of results from the individual experiments generally confirmed core features of specific stress responses in plant shoot tissue as described in comparable studies. Low night temperature, low N and low P stress, all caused significant changes in the plant phenotype. Leaf elongation as well as biomass accumulation was reduced by stress. N deficiency caused decreases in transcripts directly involved in nitrate reduction. Furthermore, the majority of primary C and N metabolism, especially C assimilation, the oxidative pentose phosphate pathway and amino acid synthesis were down-regulated (Figure 5). Amino acid and organic acid concentration in the leaves decreased while selected carbohydrates, such as starch or raffinose, and secondary metabolites increased (Figure 3; [22,23,25,26,41]). A metabolic shift towards secondary metabolites was also observed under P starvation conditions (Figure 3), but in contrast to low N stress, amino acids accumulated under low P conditions while phosphorylated intermediates decreased (Figure 3; [13,42]). P starvation was also characterized by initiation of a specific set of transcripts (phosphorus starvation induced) which included phosphate transporters, SPX-domain proteins and sulfolipid metabolism related genes (Figure 7, Additional file 5: Table S4; [13,27]). Low temperature stress applied to plant leaves in the light resulted in photodamage and an increase in the ROS detoxification system [16]. Limitation of cold stress to the dark period can avoid serious damage to the photosynthetic apparatus [34], but many other features are generally shared between light and dark chilling stress such as reduction in transport processes (Table 5) and the accumulation of amino acids, organic acids, phosphorylated intermediates, sugars and starch (Figure 3; [14,16]).

Comparison of microarrays from heat, cold, darkness, desiccation, salt and UV-B stressed maize leaves had been dominated by transcriptional changes into same direction, revealing general abiotic stress markers [43]. Contrary to that, metabolites and transcripts significantly altered by the stress conditions in our experiment were characterized by patterns with opposite reactions in at least two of the experiments; and very few features showed a common response. Only 54 transcripts and four metabolites changed significantly in the same direction (Tables 4 and 6). From the primary metabolic pathways inspected in more detail, nitrate reduction generally decreased under stress, and cellulose synthases were generally induced (Figure 5).

The general transcript and metabolite profiles reacted in an antagonistic way especially to low N and low P treatment. They were separated by the same principal component in opposite directions from the controls (Figure 1). On metabolite level, the differences were reflected in opposite changes in amino acids and phosphorylated intermediates. The synthesis of these compounds was directly affected by limitations of N or P respectively. Additional differences were observed for several carbohydrates including starch. These accumulated under low N, but were negatively affected by low P. Under low temperature stress, almost all measured primary metabolites accumulated, and therefore had effects similar to low P samples for amino acids, and to low N stressed samples for phosphorylated metabolites and other carbohydrates (Figure 3). Remarkable was the carbohydrate starvation response observed specifically under low P conditions in the maize leaves. This contrasts with the situation described for many other abiotic stress experiments including P deficiency [12,44]. The conflict is probably connected to differences in duration and/or severity of the stress [30]. In maize, short-term P stress caused accumulation of sugar and starch, but prolongation of the stress lead to depletion of carbohydrates [45]. The conditions in our low P experiment seemed therefore harsher than in many comparable studies. Since the majority of studies had been done in other plant species, this could also indicate an especially high sensitivity of maize to low P stress. The comparably low number of general stress marker in our study could also be connected to the extreme situation of carbohydrate and energy metabolism in the low P treated maize leaves.

Similarities and differences in regulation of primary metabolic pathways under low temperature, low N and low P stress related disturbances will be discussed in more detail below.

### Ion balance of stressed leaves is regulated in a nutrient specific manner

Deficiencies of N or P generally impact on the uptake activity in the root and the associated transport systems. Since the root is also responsible for acquisition of all other nutrients, metabolism of all ions needed to be adjusted. The major nutrients P, S, K, Ca and Mg were significantly changed under low temperature, low N and low P in an element specific manner (Table 3), supporting the presence of active control mechanisms for their uptake and allocation [46].

Potassium (K) concentrations increased under all stress conditions indicating that its uptake was not considerably impaired by the stress. In plant material, K represents the most abundant cation, it contributes to enzyme activation, protein stabilization, osmo-regulation, turgor adjustment and ion transport regulation [47]. A similar strategy seemed to be present for P; its concentrations increased under cold and low N conditions, only under low P conditions its accumulation was limited by supply (Figure 7). P accumulation was particularly strong in low N treated plants, and there are indications, that phosphate uptake is actually promoted under low N conditions. Under low P conditions, the transport of sucrose from the shoot into the root increases and stimulates the phosphate transporters for improved uptake [12,48]. The sucrose related increase in phosphate uptake could also be active under low N stress conditions, because root growth is promoted by P as well as N deficiency [11]. Additionally, phosphate uptake in Arabidopsis seems to be enhanced under low N conditions via a signaling pathway involving miRNAs and the E3 ligase NLA (nitrogen limitation adaptation) [49]. The accumulation of K and P, underlines the importance of both elements for plant metabolism and growth, and it seems economically advantageous to transport and store them in the leaves.

In contrast to the excess uptake strategy of P and K, N did not accumulate significantly under low temperature and low P stress conditions. Therefore N economy of the leaf seemed to be regulated by demand rather than availability. A concomitant decrease in abundance of nitrate metabolism related transcripts indicated a general down-regulation of N assimilation under low temperature and low P stress (Figure 6). Under conditions of N limitation, the total N concentration decreased in the leaves and the same behavior was observed for sulfur (S). S is integrated into proteins, and therefore S metabolism is tightly linked to N metabolism. The metabolism of both elements correlated to decreased demand of amino acid synthesis for growth processes under stress [50].

The decline in Mg and Ca concentrations under stress suggested similar control strategies for these elements. Mg forms part of the chlorophyll complex and its utilization diminishes when synthesis of phytylesters is reduced (Table 4). Most minor nutrients also seemed to be regulated by demand rather than availability under stress. The decrease of Mo was particularly pronounced under low N conditions and since Mo serves as a cofactor for nitrate reduction, the very low Mo levels could reflect the strong reduction in nitrate assimilation. The situation is similar for Fe which is also involved in several steps of nitrate metabolism [51]. In contrast to minor nutrients, concentrations of trace elements tended to increase under stress. It is possible that the control of their uptake is under less strict control and the increase is simply a result of continued uptake during retarded growth under stress.

### Phosphorus deficiency causes very distinct disturbances in carbon assimilation and energy metabolism

The majority of abiotic stress conditions do not affect the primary carbon assimilation reaction directly. The decreased demand of photosynthates for biomass production usually causes accumulation of carbohydrates in the leaves and feed-back inhibition of photosynthetic gene expression and finally C fixation [52,53]. In our experiments, low temperature and low N treated plants followed this established pattern. Transcripts related to the two photosystems, the C_4_ shuttle and the Calvin-Benson cycle were reduced in the stressed plants in a coordinated manner (Figure 5; Additional file 5: Table S4). Especially in the low temperature treated leaves, transcripts for the PSI and PSII decreased and this might be directly connected to the higher sugar accumulation under these conditions. The photosynthetic gas exchange was not affected in the cold treated plants, it even showed slightly enhanced values, which could be connected to an increased density of Calvin-Benson cycle enzymes in leaves grown under low temperature condition [54]. Leaves of low N grown plants usually save N by reducing synthesis of chlorophyll and proteins [55]. In the N experiment, C assimilation in low N treated plants was lower than in the controls. Accumulation of phosphorylated intermediates indicated that downstream C utilization was reduced and contributed to the feedback inhibition.

The situation in low P grown plants seemed more complex. Prolonged P starvation led to depletion of phosphorylated intermediates, carbohydrates, organic acids and sugar as well as starch levels (Figure 3). Under these conditions, photosynthesis was more likely to be restricted directly by phosphate limitation of photophosphorylation and associated C metabolism [56], because P deficient leaves suffer from shortage of ATP and reduced adenylate energy charges [57]. The different regulation of C assimilation under low N and low P stress also seemed to have consequences for stomatal behavior. Stomata control leaf gas exchange by balancing CO_2_ supply for photosynthesis against water loss [58]. The reduced demand of CO_2_ under low N conditions was therefore adjusted by a decrease in stomatal conductance, and the internal CO_2_ concentration (Ci) remained constant in stressed and control leaves (Table 2). The P limitation related decrease of C assimilation on the other hand did not affect stomatal conductance in the same way, leading to an increase in the calculated Ci of the maize leaves (Table 2). A similar discrepancy between P starvation effects on C assimilation and stomatal conductance had already been observed for P starved sunflower, wheat and maize in an earlier study by Jacob and Lawlor [59]. In the field, changes in stomatal control by extreme P limitation thus could have serious consequences for the water use efficiency of the plants.

Carbohydrate feedback usually leads to decreases in abundance of photosynthesis related gene expression [60], and this could also be observed in the low temperature and low N treated leaves of our experiments (Figure 5, Additional file 5: Table S4). In the P experiment, however, gene expression of PSI and PSII related transcripts was enhanced. The same phenomenon was observed in extremely P limited maize plants growing under low P supply and in absence of arbuscular mycorrhiza; their promotion of gene expression was not only observed for the photosystems but also for C_4_ and Calvin-Benson cycle related transcripts (N. Zellerhoff, personal communication). It is possible, that the low sugar concentration in the leaves was responsible for the induction or at least de-repression of photosynthesis genes. The increased expression could also be connected to a higher turnover of photosystem related proteins and transcripts, caused by overexcitation and photodamage. The accumulation of metabolites with protective function such as lutein or zea- and antheraxanthin (Figure 3) also indicated enhanced demand for protective systems especially under low P conditions.

Phosphate is not only involved in the initial photophosphorylation, but also in downstream reactions of C_4_ photosynthesis [61]. In the C_4_ shuttle mechanism, CO_2_ is initially bound to PEP by the PEPC in the mesophyll producing oxaloacetate which is further converted into malate and transported to the bundle sheath for CO_2_ release in the proximate environment of Rubisco. It had been shown for C_3_ plants, that the carboxylation of PEP increases under low P conditions, thus bypassing the pyruvate kinase reaction under limited Pi and ATP conditions [62]. In the maize leaves of the P experiment, the housekeeping PEPC transcript increased in expression about 14 fold (Additional file 6: Table S5), such an increase was absent for cold treated leaves and the transcript decreased under low N (Additional file 6: Table S5). If P deprivation affected the availability of PEP, this could impair the operation of the C_4_ shuttle and the ratio between oxygenation and carboxylation reaction of Rubisco. Data from C_3_ plants actually suggested a general increase of the photorespiratory pathway under low P conditions, and it has been postulated that the release of phosphate during hydrolysis of 2PG contributes to the phosphate balance in the chloroplast [62]. The photorespiratory amino acids glycine and serine increased specifically in the P deficient maize leaves, but the organic acids glyoxylate and glycerate were not affected (Figure 3). Since glycine and serine are also involved in the C1 metabolism, the present data is insufficient for general statements about the activity of photorespiration in C_4_ plants under low P conditions. It would however be worthwhile to investigate the specific influence of P deficiency on C_4_ photosynthesis in more detail.

The P stress related problems in synthesis of energy equivalents would also affect further cellular reactions. In the maize leaves, this was visible by transcriptional induction of several genes involved in alternative pathways, e.g. PPi-dependent phosphofructokinase (PFK), PEPC, fermentation and alternative oxidase (Additional file 4: Table S3). The situation showed thereby similarities to the energy crisis suffered in plants under low oxygen supply (see reviews: [63,64]). Pyrophosphate concentrations in the cytoplasm are usually less sensitive to P deficiency than phosphate and ATP levels, and induction of a PPi-PFK transcript would indicate the rerouting of specific catabolic steps under low P conditions. Interestingly, the same transcript was regulated into the opposite direction under low temperature and low N conditions. Additionally, low P treatment was characterized by transcriptional up-regulation of AOX (Additional file 4: Table S3), a bypass reaction to the oxidative phosphorylation [65]. Their increased activity would allow continued functioning of TCA cycle and electron transport chain with limited ATP productions and prevent oxidative damage [66]. Generally, the shifts in energy metabolism related transcripts confirm that the leaves from low P treated plants suffer from ATP shortage. Absence of similar responses or even opposite reactions indicated that energy supply was not limiting under low temperature or low N conditions.

### C partitioning is characterized by a shift towards starch under cold and low N stress, but a preference for sucrose under low P stress

C assimilated in the photosynthetically active leaf is primarily synthesized into sucrose for transport into sink organs or incorporated into starch as an internal C reserve. Starch synthesis increases, when the rate of CO_2_ fixation exceeds the demand for sucrose synthesis and export (see review: [67]). In the stressed maize leaves, changes in starch concentrations mirrored the general C status of the leaves. Low temperatures had no marked effect on C assimilation rate, so growth retardation and reduction in transport processes led to significant increases in sucrose and starch (Figure 3). In N stressed leaves, C assimilation was slightly affected, but enhanced amounts of C were synthesized into starch. The results generally confirmed promotion of starch synthesis under cold [16], and low N stress [22,23]. The response was different in the C starved low P treated leaves. Under these conditions, the assimilated C is preferentially partitioned in to sucrose [45].

These preferences for C partitioning were reflected in differences of transcriptional regulation of sucrose metabolism (Figure 5, Additional file 5: Table S4). In low temperature and low N treated leaves, sucrose synthesis and transporter genes were down-regulated, indicating that repression of sucrose metabolism contributed to starch accumulation. No such transcriptional repression was observed under low P conditions (Figure 5, Additional file 5: Table S4). In contrast to C_3_ plants, synthesis of sucrose and starch in the mature C_4_ leaf is localized in different cell types. Starch accumulates almost exclusively in the bundle sheath while sucrose synthesis takes place in the mesophyll [68]. The stress related shift towards one of these endproducts would therefore affect the metabolism of both cell types in a different way. Sucrose and starch synthesis both play important roles in the cellular recycling of phosphate for continuation of photosynthetic reactions. The control of phosphate homeostasis in C_4_ leaves might therefore require regulation of phosphate distribution between cell organelles as well as phosphate balancing between mesophyll and bundle sheath cells [69].

Independent of the nature of the stress, the amounts of transcripts for cellulose synthases were enhanced in all stressed maize leaves and therefore correlated negatively to the growth behavior of the plants (Figure 5). This could indicate generally increased partitioning of C into the cell wall under stress. However, cell wall formation continues even after the leaves have reached the final size, and it is possible that slight developmental differences in the control and stressed plants contributed to changes observed for transcription of cellulose synthesis. In monocot species, such as maize, developmental changes can be followed along the leaf gradient [70,71]. Transcripts involved in leaf maturation would be present in high amounts in the base and decrease towards the tip. The expression of cellulose synthases and other transcripts with predicted function in plastid maturation such as RS2 [37] followed this pattern in the maize leaf (Additional file 2: Figure S2). It can therefore not be excluded that stress related retardation of the growth program influenced the measured expression pattern. Analysis of stress related changes should therefore generally be viewed critical with respect to their growth patterns.

### Nitrate and amino acid metabolism adjust to demand under low temperature, low N and low P stress

During the day, a large proportion of nitrate is transported from the root to the shoot, where it is reduced and assimilated into amino acids and other organic compounds. In contrast to management of P, total N concentration did not increase significantly in the low temperature or low P stressed leaves (Table 3). Nitrate allocation within the plants depends on the activity of various specific transporters [72,73]. After uptake into the cell, nitrate is reduced to nitrite in the cytoplasm by nitrate reductase, followed by nitrite reduction in the chloroplast by nitrite reductase to ammonium (Figure 6). In the maize source leaves, the expression of many nitrate transporters, nitrate reductases and nitrite reductase was reduced by low temperature, low N and low P stress (Figure 6, Additional file 5: Table S4). Expression of nitrate assimilation related transcripts therefore showed positive correlation to the general growth rate of the plant, and it can be postulated that the reactions were regulated by demand of reduced nitrate for synthesis of building blocks for biomass. Nitrate assimilation requires considerable input of energy and reductants from the cellular metabolism [74]. Reduction in its activity would liberate this power for stress adaptation.

The activity of nitrate reductase is tightly regulated on transcriptional, post-transcriptional and enzyme activity levels [75]. Different control mechanisms should, however, have been active under the different stress conditions (Figure 6). Expression of nitrate reductase can be regulated directly by substrate (nitrate) supply [22]; and under low N conditions, nitrate limitation should have influenced transcript abundance. Under low temperature and low P stress, nitrate should have been available in abundance; and under these conditions, amino acid (end-product) accumulation would repress nitrate reductase transcripts [76,77]. Higher amino acid accumulation under low P thereby corresponded to stronger repression of nitrate metabolism related transcripts (Figure 6). The low carbohydrate levels in low P stressed plants could additionally negatively influence nitrate reductase expression [78]. Since the advantage for repression of nitrate reduction under stress lies mainly in the saving of energy and reductant, this strategy would also be more relevant for leaves under P starvation than under low temperature. The decrease in expression of several nitrate transporters indicated that also the allocation of nitrate to the shoot was negatively regulated in stressed leaves (Figure 6; Additional file 5: Table S4). Generally, the very tight regulation of nitrate assimilation under very different metabolic stress conditions confirmed the importance of its control for stress adaptation. Besides low temperature, low N and low P stress, repression of nitrate assimilation had also been observed under drought stress [79].

Nitrate assimilation produces ammonium, which is further incorporated into amino acids by the GS/GOGAT cycle. In contrast to the nitrate reduction process, transcripts for GS and GOGAT were much less affected by the different stress conditions (Figure 5, Additional file 5: Table S4). In the cell, ammonium is not only produced by primary nitrate fixation but also from photorespiration and turnover of amino acids and proteins [80]. Immediate re-fixation of ammonium under stress would avoid loss of reduced N to the environment, which is important especially under N deficiency [81].

The majority of amino acids followed a similar pattern under the different stress condition: slight increase under low temperature, decrease under low N and considerable increase under low P conditions (Figure 3). Correlation between amino acid changes and expression of amino acids related transcripts was however limited. Transcriptional regulation had been shown for asparagine metabolism [82]. With a high N:C ratio the amino acid can serve as N storage compound, under low N conditions specific transcripts involved in its synthesis decreased while transcript involved in its degradation increased [26]. The opposite picture could be observed under low P conditions, when N is available in abundance, but C skeletons were limited due to general shortage of carbohydrates. Another example for correlation between amino acid concentration and levels of related transcripts could be found for proline. The final enzymes of the proline synthesis pathway were strongly induced in cold stressed leaves and this correlated with a particularly strong accumulation of proline (Figure 3, Additional file 5: Table S4). This specific amino acid serves as protectant of cellular structures in different abiotic stress situations [83], including low temperatures and our results confirmed that this accumulation was influenced by transcriptional control. Under low N and low P condition, no specific proline responses could be observed, showing that proline accumulation is not a general stress response but regulated only under specific environmental conditions.

N metabolism is strongly interconnected with C metabolism. Organic acids for instance supply C skeletons for amino acid synthesis and C partitioning into the organic acid pool is also supposed to be regulated by N supply [77]. Such a correlation between amino acids and organic acids was present in the maize leaves under low temperature and low N treatment. The level of compounds from both groups increased under low temperature and decreased under low N. The correlation was, however, lost under low P conditions (Figure 3). Their general C shortage resulted in reduction of organic acids, while amino acids accumulated [42]. Amino acid accumulation resulted more likely from a decline in protein biosynthesis. Only in low P treated leaves, a coordinated reduction in transcripts for ribosomal proteins was observed (Figure 5, Additional file 5: Table S4) indicating general repression of protein synthesis under P starvation. In comparison with maize seedlings grown under P abundance, P starved leaves usually also had decreased total protein contents [84]. Repression of protein synthesis probably represented another energy saving adaptation process under P limitation.

In summary, the maize source leaf metabolism adapted to N limitation by reduced input into nitrate assimilation, but also transcriptional down-regulation of many other pathways of primary metabolism (Figure 5). Supply of stressed plants with abundant N did not initiate the buildup of N storage pools.

### Control of phosphate homeostasis under stress depends on transcriptional regulation of a specific set of genes

The strategy for adaptation to variation in P supply followed a very different strategy. Abundance of P caused accumulation of the element in the leaves. The concentration of P in the leaf tissue correlated negatively to a specific set of significant transcripts. About 40% of these transcripts had assigned functions in P salvation and transport processes. Transcripts with the same expression patterns but so far unknown function could represent further candidates for regulators of P homeostasis (Figure 7, Additional file 6: Table S5).

An important P salvation mechanism involves the replacement of phospholipids in the cellular membranes by sulfo- and galactolipids. The synthesis of sulfolipids was therefore promoted under low P conditions by an increase in the SQD2 transcript. Arabidopsis mutants without a functioning SQD2 protein showed retarded growth under P deficiency supporting the importance of sulfolipid in the adaptation response [85]. In the examined maize leaves, the SQD2 homolog increased significantly under low P stress, but the transcript decreased significantly under low N and cold (Additional file 5: Table S4). Under conditions of high phosphate availability, incorporation of sulfur was therefore actually repressed and by this way incorporation of P seemed to be actively promoted. Transcript patterns of several purple acid phosphatases also indicated increased activity under low P conditions and subsequently release of phosphate from phosphoesters [86], while decreased abundance of the same transcripts under low temperature and low N suggested reduced phosphatase reaction (Additional file 5: Table S4).

Distribution of P needs to be regulated in the whole plant as well as at the cellular level [87,88]. After phosphate uptake in the root, it is released into the xylem and transported into the shoot. Phosphate demand is thereby highest in young tissue, and older leaves contribute to phosphate supply of growing tissue. Since maintenance of root metabolism is vital for whole plant survival under nutrient deficiency, phosphate can also be redistributed from the shoot to the root [74,89]. Within the cell, many processes are dependent on phosphate distribution between organelles. Excess of phosphate in the cytosol could for instance drain the chloroplast of triose-P [90]. Stabilization of phosphate homeostasis can be conferred by buffering the system through exchange with the phosphate pool in the vacuole [88]. Features involved in the phosphate control system should therefore include mechanism avoiding excess as well as shortage of phosphate in the metabolically active compartments. This correlates with the identification of numerous transcripts related to P metabolism with opposite expression patterns under conditions with increased phosphate levels under low temperature or low N, and decreased levels under low P (Figure 7).

In Arabidopsis, various transcripts involved in regulation of P metabolism have been identified. These include the transcription factor PHR1 and the phosphate control system consisting of the E3 ligase SIZ1, miRNA399, the E2-conjugase PHO2 and At4/AtIPS1 (for review see: [91,92]). Homologues of the Arabidopsis genes could be identified in other plant species such as rice and maize [38] indicating evolutionary conservation in the phosphate regulatory mechanism. In the maize leaves, expression of the At4/IPS1-like RNA showed a very strong negative correlation to phosphate concentration of the leaves under all tested stress conditions (Figure 7). The PHO2-like transcript on the other hand was only slightly reduced by low P; and surprisingly a much stronger down-regulation was observed under low temperature and low N, when phosphate concentration was high. In Arabidopsis PHO2 down-regulation of inhibited remobilization of phosphate from older to young shoot tissue [89]. Its strong regulation in the maize leaves could indicate that phosphate remobilization was down-regulated to a greater extent in phosphate abundant than in phosphate deficient leaf tissue, thus promoting retention of phosphate in leaves under generally high P conditions.

Four groups of phosphate transporters have been described in Arabidopsis [93]. Best studied are high-affinity transporters from the PHT1 group, they have been localized in the plasma membrane and mediate phosphate uptake in the roots under P limiting conditions [94]. PHT2-like low affinity phosphate transporters are associated with chloroplasts, while the PHT3 transporters are predicted to be mitochondrial phosphate carriers. Different members of the PHT4 groups have been localized in the plastid envelope or the Golgi membrane system. Knowledge about phosphate transport system across the tonoplast, which would be important for the exchange of phosphate between cytosol and vacuole, is so far limited to kinetic studies [95].

In the maize leaves, expression pattern of PHT1-like high affinity phosphate transporters correlated negatively with phosphate concentrations (Figure 7). Considering the plasma membrane localization of this transporter group, the results suggested that uptake of phosphate by leaf cells was transcriptionally down-regulated under high P conditions, and up-regulated under P deficiency (Figure 7). Expression patterns of transcripts from the other groups (PHT2-PHT4) showed much less correlation to the soluble and total P concentrations measured in the maize leaves (Figure 7; Additional file 5: Table S4). Their regulation was probably more dependent on phosphate levels in the organelles than on total P concentrations in the leaf.

Another group of proteins usually connected to phosphate transport are SPX (SYG1/PHO81/XPR1) domain proteins (see review [96]). In yeast, SPX proteins have been described for their involvement in control of intracellular phosphate homeostasis and also for their contribution to vacuolar transport [97]. In plants, SPX domain proteins are also connected to phosphate transport or at least in its fine-tuning, but their precise mode of action is still unknown [96,98]. SPX domain proteins in plants are further divided into subgroups depending on the existence of additional domains (SPX; SPX-EXS; SPX-MFS; SPX-RING). In the maize leaves, different SPX domain proteins showed strong P status dependent expression patterns supporting an involvement in control of intracellular phosphate homeostasis in leaves (Figure 7).

Studies in Arabidopsis and rice indicated that the different SPX domain proteins interact with each other, forming a regulatory network depending on activating and inhibiting interactions [96,98]. The results from maize would also support interaction between different SPX domain containing proteins. Simple SPX domain proteins were thereby regulated in coordination, while an SPX-MFS protein showed a differential picture (Figure 7). In Arabidopsis, a SPX-MFS proteins had been assigned to the vacuolar membrane [99] and the maize homologues therefore represent good candidates as regulators of phosphate exchange between cytosol and vacuole under changing P regimes [100]. In the suggested system, the SPX-MFS transcript with increased expression under high P status could be responsible for removal of phosphate from the cytosol. A related transcript showed the opposite pattern, and it is possible that vacuolar import and export of phosphate are controlled by different proteins. The SPX-MFS transcript with reduced abundance under high P was not affected by low P conditions, so it might be involved in reduction of phosphate transport out of vacuole when phosphate is generally high, but does not influence phosphate export under low phosphate conditions (Figure 7).

The so far best characterized SPX domain containing protein is PHO1 (SPX-EXS domain protein) and there is evidence for its function as an exporter of phosphate from the cell [101]. Overexpression in Arabidopsis leads to drainage of Pi from the leaf cells into the apoplast [102]. The maize homolog of PHO1 was down-regulated under low temperature and low N conditions, when P status of the tissue was high, thus also supporting intracellular storage of excess phosphate and reduction of phosphate transport out of the cell. The results further supported a reduction in phosphate remobilization from leaf cells into other plant parts when the general phosphate status is high.

The phosphate homeostasis regulatory system is further influenced by sucrose concentrations. Increases in sucrose thereby enhance the induction of SPX proteins and other phosphate starvation response genes [94]. The P starved leaves had generally low carbohydrate contents, but the above described preference for C partitioning into sucrose would support the induction of SPX domain proteins. The presented results would suggest that phosphate balancing plays a major role in stress adaptation, especially under conditions which affect P as well as C status of the plant. Disturbances in regulation of P homeostasis, e.g. by mutation of a phosphate regulator such as PHO2, can therefore interfere with the plants adaptation capacity to stress [103].

## Conclusions

Maintenance of cellular metabolism under stress depends on stabilization of all metabolic processes. Exposure of maize leaves to prolonged low temperature, low N and low P conditions caused very different disturbances in the primary C, N and P metabolism. The selection of stress conditions with contrasting effects on specific metabolic processes helped in understanding their regulation.

Regulation of central parts of C metabolism showed similarities under low temperature and low N stress. The stress related growth retardation was thereby connected to accumulation of carbohydrates including starch thus indicating that growth was not limited by C assimilation, but that photosynthesis was feed-back regulated. Simultaneously, transcripts associated with C assimilation and sucrose synthesis were reduced. In contrast to this, P deficiency influenced C and energy metabolism in a more direct manner. Photosynthetic activity decreased considerably, carbohydrates and related metabolites declined and alternative energy processes were induced. The results underlined the importance of optimal phosphate balance for efficient photosynthesis. In the C_4_ plant maize, the CO_2_ concentrating shuttle mechanism could also be affected. The lack of carbohydrate stores would impact negatively on further plant development and finally yield.

Transcripts associated with primary nitrate metabolism were considerably decreased under all tested stress conditions. Nitrate assimilation thus seems to be regulated by demand and not by availability, and different control systems should have taken effect under conditions of limiting and abundant nitrate supply. The transcript data suggested close correlation between activity of nitrate assimilation and the plant’s growth rate. Because the plants do not build up stores in the leaves, their growth would always be highly dependent on the availability of N in the soil.

The plant’s strategy was very different for long-term management of P. Abundant availability of P stimulated accumulation of P stores in the leaves. A cluster of transcripts with very strong correlation to P concentrations in the leaves could be identified. Several of these had been described for their involvement the P starvation response before, but the list also provided new candidates with possible function in regulation of P homeostasis.

## Methods

### Plant growth

Maize seeds (*Zea mays* cv. B73) were submitted to the different stresses in independent experiments. Each experiment consisted of control plants and stressed plants. The low temperature experiment was conducted in autumn 2009 in two successive experimental sets. N deficiency tests were carried also carried out in two successive experimental sets in spring 2010. P deficiency was applied to one experimental set in summer 2011. Photosynthesis measurements were performed in additional independent experiments for low temperature and low N tests. Plants used for photosynthesis analysis of the nutrient experiments were thereby cultivated at the same time in the same growth chamber, and this happen in parallel to the initial P experiment.

In the low temperature experiment the seedlings were subjected to cold stress in the dark period only. In the nutrient deficiency experiments, seedlings were supplied with nutrient solutions containing either insufficient nitrate or insufficient phosphate for optimal growth. For the nutrient experiments, control plants were cultivated under exactly the same conditions, but experimental variation in growth could still be observed. The low temperature experiment was arranged in a different growth chamber which allowed setting night temperatures down to 4°C. The use of different growth facilities also caused variation in light quality and quantity during seedling growth.

Low night temperatures had the strongest effects on early seedling growth, and 20 days after germination the biomass of stressed plants was already reduced to ca. 40% of the control plants. A comparable stress severity was achieved by low N or low P stress only 30 days after germination. In order to be able to compare stress conditions causing similar growth retardation, plants from the low temperature experiment were harvested at 20 days after germination, plants from the nutrient stress treatments were harvested at 30 days after germination. The analysis of results concentrates on the differences between stressed plants (low temperature, low N or low P) and the associated controls (control T, control N and control P).

For all experiments, seeds were incubated on wet filter paper for three days, and uniform seedlings were transferred into pots of 1.5 L volume containing nutrient poor peat soil (Basissubstrat 2, Klasmann & Deilmann, Germany). Fertilization started at day seven of the experiment with modified Hoagland solutions. Control and low temperature stress treated plants were supplied with 15 mM KNO_3_, 5 mM CaCl_2_, 2 mM MgSO_4_, 2 mg/L Fe, 0.5 mM KH_2_PO_4_, 50 μM H_3_BO_4_, 10 μM MnCl_2_, 1 μM ZnSO_4_, 0.3 μM CuSO_4_, 0.5 μM Na_2_MoO_4_. For low N treatment, the nitrate content was reduced to 0.15 mM KNO_3_, for low P treatment the phosphate content of the nutrient solution was 0.1 mM KH_2_PO_4_. The differences in potassium supply were balanced with KCl. Plants received 100 ml of nutrient solution every third day, between the fertilisation supply, plants were watered with distilled water depending on water status in the pot. The last watering with nutrient solution always happened two days before the harvest. Plant growth performance was assessed by daily measurements of leaf length from ligule of the most previous developed leaf until tip. For the leaf elongation rate data in Table 1 measurements from at least four consecutive days before harvest were used.

The low temperature experiment was performed in two Snijders climate chambers (IMAGO F3000, Snijders Scientific) at 14 h days with 28°C and 80% humidity, followed by 10 h of night at 20°C and 50% humidity. Light intensity on soil level was ca. 600 μmol m^-2^ s^-1^, and ca. 800 μmol m^-2^ s^-1^ at the position of the highest leaves. All seedlings were allowed to establish in the pots under control conditions. At day 7 after germination, conditions in one chamber were switched to low temperature stress and the night temperatures were reduced to 4°C while all other settings were kept constant. At day 20 after germination leaf five represented the main source leaf and it was harvested for analysis of ion, transcript and metabolites profiles.

The nutrient deficiency experiments (N, P) were performed in a CLF climate chamber (PlantMaster PGR 3045, CLF Plant Climatics GmbH, Germany) with a diurnal rhythm of 14 h light (ca. 200 μmol m^-2^ s^-1^ at level of the soil surface and ca. 650 μmol m^-2^ s^-1^ just under the light source which was adjusted according to plant height to give maximal possible irradiance) at 28°C and 80% humidity, and 10 h night at 20°C and 50% humidity. The nutrient deficiency stressed plants received modified nutrient solution for the whole period of the experiment and at 30 days after germination, growth retardation in stressed plants was comparable to the cold experiment. Leaf six was harvested as a source leaf in all plants for further molecular analysis.

Harvest of plant material always started two hours after start of the light period and was always completed before midday. The whole plant was cut at soil surface and immediately weighed for shoot fresh weight analysis. The leaf lamina was harvested 2 cm above the appearance point and the top 6 cm from the tip were removed. The remaining mid-part of the leaf was immediately frozen in liquid nitrogen and stored at −80°C. The whole leaf was homogenised with cooled mortar and pistil and aliquoted under liquid nitrogen for transcriptome and metabolome and ionome anaylsis. A subsample of leaf and stem material was dried at 80°C for 3 days for determination of plant dry weight. Since there was no significant difference in fresh/dry weight ratio of stressed and control plants, biomass of plant is usually given as fresh weight.

### Photosynthetic parameter

Gas exchange and PSII fluorescence were determined in the main source leaves (leaf 5 in low temperature, leaf 6 in low N and low P experiment) using a combined infrared gas exchange-chlorophyll fluorescence imaging system (GFS-3000 and Mini-Imaging PAM fluorometer; Heinz Walz, Germany) at actinic illumination of 600 μmol m^-2^ s^-1^, 28°C leaf temperature, 400 mL L^-1^ CO_2_ and 13,000 mL L^-1^ H_2_O. Assimilation, conductance and internal CO_2_ concentration were calculated according to Farquahr et al. [104], effective PSII quantum yield was determined after Gentry et al. [105]. Because already small injuries of the plants can influence molecular parameter, the photosynthesis measurements were always done on maize seedlings from different sets of experiments. Plants for analysis of photosynthetic parameter under low N and low P stress were grown at the same time in the climate chamber and data for control N and control P is therefore identical.

### Ion content analysis

For determination of different elements by ICP-MS analysis a microwave digestion system (Anton Paar, Multiwave 3000) was employed. Approx. 0.5 g of fresh, homogenized plant material was digested using 4 ml of concentrated HNO_3_ (66%) and 2 ml of H_2_O_2_ (30%). Final solutions were diluted with deionised water to an end volume of 30 ml. The reagent blank was treated in the same way. The program used in the microwave starts with a power ramp of 10 min followed by 30 min at 1400 W and finished with 15 min of cool down. As a certified reference material “hay powder” was used. The concentration of 21 elements was determined using an Agilent 7700 ICP-MS (Agilent Technologies, Tokyo, Japan) following the manufacturer’s instructions. Five biological replicate samples were used for ion profile analysis. Additionally aliquots from the same leaf samples were used for determination of total N and C concentrations. For this the leaf material was freeze dried, and 10 mg samples were subjected to elemental analysis using the Vario EL cube (Elementar Analysensysteme GmbH, Germany) in the CN operation mode.

### Microarray analysis

RNA was extracted from four biological replicates for each stress condition (low temperature, low N, low P) and each associated control (control T, control N, control P) described by Logemann et al. [106] using 100 mg of frozen leaf material. The isolated RNA was purified with the RNeasy purification kit according to the manufacturer’s instructions (Qiagen, Germany). The quality was checked with the Agilent 2100 Bioanalyzer using the RNA 6000 Nano kit. The cDNA and following antisense cRNA synthesis was performed according the one-color microarray-based gene expression analysis protocol (Agilent Technologies). An aliquot of 1.65 μg of Cy3 labeled cRNA was loaded on one-color microarrays with custom-designed oligonucleotide probes (Agilent 025271). Randomization of samples from low temperature and low N experiments over different chip batches and hybridization dates had shown that this had no marked influence on results; samples from the P experiment were hybridized in one set. Validation of data for the described microarray method by quantitative RT-PCR was demonstrated by Pick et al., [71]. Transcripts were normalized to the 75^th^ percentile followed by baseline correction across all samples using the Agilent Gene spring GX12.0 software. The sample from the low temperature and low N experiments had been randomized using different chip batches and different hybridization dates, but the influence of these two parameters was judge as minor and samples from the low P experiment were measured in one batch. Annotation and classification followed the characterization of the Agilent 025271 array chip as described in the OPTIMAS databank [107]. Transcripts from the primary C, N and P related pathways presented in the paper were manually confirmed. For this, orthologs of the transcripts of interest from Arabidopsis and/or maize were selected from databases (TAIR, NCBI, MaizeGDB) and blasted against the sequences available on the chip [107]. The functional annotation of the selected sequences was then again confirmed by another round of BLAST search in the NCBI database. In the summarized data only the data from one representative signal output is shown when multiple identifiers were present for the same transcript (alignment according to the 4a.53 B73 genome assembly). All array data can be accessed under submission number GSE46704 in the NCBI Gene Expression Omnibus database. The data for the N experiment had already been used in a previous paper by Schlüter et al., [26].

### Metabolites

Lyophilized tissue equivalent to 200 mg of fresh weight was used for metabolite profiling. Metabolites were extracted with the use of accelerated solvent extraction with polar (methanol + water, 80 + 20 by volume) and nonpolar (methanol + dichlormethan, 40 + 60 by volume) solvents. Subsequent analyses of metabolites by gas chromatography–mass spectrometry (GC-MS) were performed as described elsewhere [108,109]. Sucrose and starch levels were determined in 100 mg of homogenized leaf material extracted with 80% (v/v) ethanol and 20 mM HEPES, pH 7.5, as described by Stitt et al. [110] but adapted for determination in a plate reader by direct downscaling the assay to a volume of 200 μL. Three to five replicate samples were measured per treatment.

### Data analysis

Statistical analysis of microarray data was performed using the Agilent GeneSpring GX12.0 program. The significance level for differently regulated feature was set at a fold change of FC > 2 and a Benjamini-Hochberg correction false discovery rate of FDR < 0.05. For metabolite and ion data, significant differences were calculated by *t*-test and cut-off level set at p < 0.05. MarkerView software 1.2 (Applied Biosystems, UK), was used for principal component analysis of transcript and metabolite data using the Pareto algorithm. GO term enrichment was performed using the AgriGO webtool [36].

## Competing interests

The authors declare that they have no competing interests.

## Authors’ contributions

Experiments were conducted and analyzed by U.Schlüter. CC and U.Scholz developed the data warehouse and provided bioinformatics tools. AB supplied annotation of the custom produced microarray and contributed to data analysis. NZ and MB provided ionomics data, HF provided metabolite data. U. Sonnewald supervised the project. The paper was written by U.Schlüter, APMW, NZ, HF and U.Sonnewald. All authors read and approved the final manuscript.

## Supplementary Material

Additional file 1: Table S1 Elemental concentrations in the maize leaf. Click here for file

Additional file 2: Figure S1 Changes in metabolites connected to primary metabolism under low temperature, low N and low P stress. Data is presented as heatmaps for log2 fold changes in stressed vs control conditions. **Figure S2** Expression of transcripts commonly upregulated under low temperature, low nitrogen and low phosphorus conditions along the leaf gradient (tip – base). Click here for file

Additional file 3: Table S2 List of metabolite peaks with significant change (*t* test: P < 0.05) under at least one stress condition. Click here for file

Additional file 4: Table S3 Transcripts with significant changes (fold change >2; FDR < 0.05) in maize source leaves stressed by low temperature, low nitrogen or low phosphorus. Click here for file

Additional file 5: Table S4 Transcriptional changes by low temperature, low N and low P in selected pathways of primary C and N metabolism, Pi homeostasis, hormone metabolism and senescence related processes. Click here for file

Additional file 6: Table S5 Intersection of significant transcriptional changes in low temperature, low N and low P treated leaves. Click here for file
