# Supplementary material for: Adaptation of maize source leaf metabolism to stress related disturbances in carbon, nitrogen and phosphorus balance
Source: BMC Genomics. 2013 Jul 3;14:442. doi: 10.1186/1471-2164-14-442 (PMC3716532; doi:10.1186/1471-2164-14-442)

Supplementary figure S1: **Changes in metabolites connected to primary metabolism under low temperature, low N and low P stress**. Data is presented as heatmaps for log2 fold changes in stressed vs control conditions.

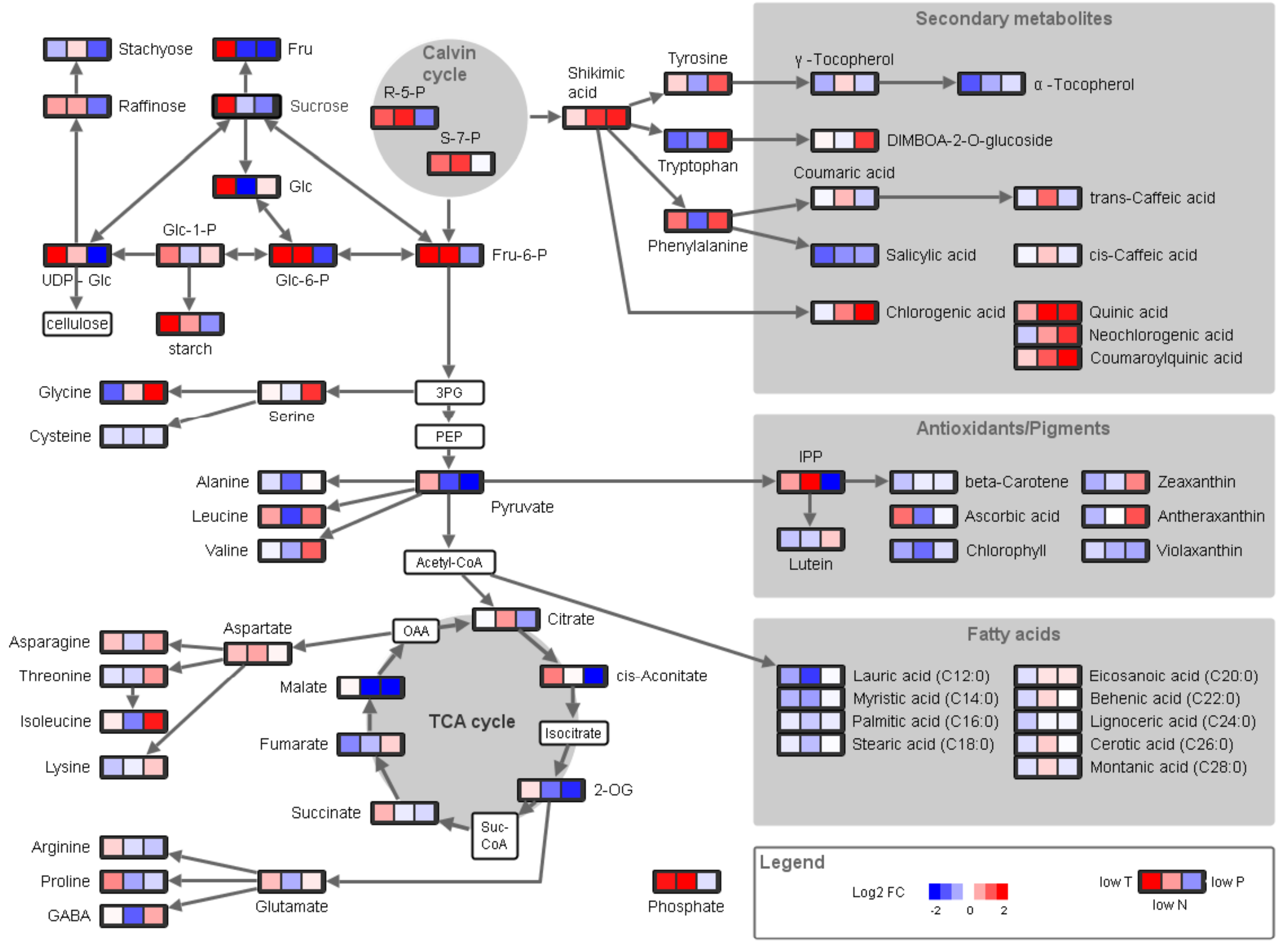

Supplementary figure S2: Expression of transcripts commonly up-regulated under low temperature, low nitrogen and low phosphorus conditions along the leaf gradient (tip – base)

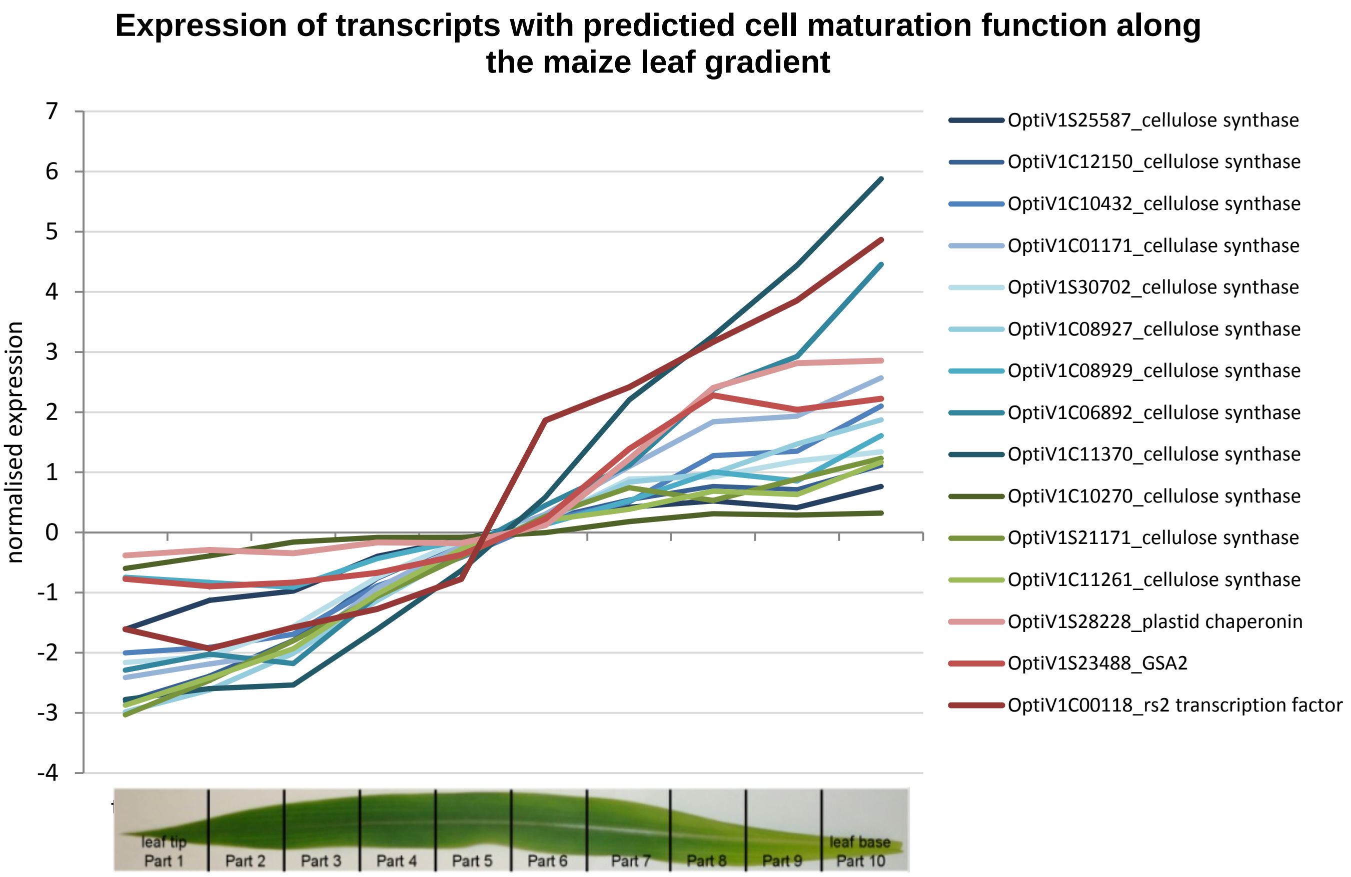

Supplement: Additional file 2: Figure S1 — Changes in metabolites connected to primary metabolism under low temperature, low N and low P stress. Data is presented as heatmaps for log2 fold changes in stressed vs control conditions. Figure S2 Expression of transcripts commonly upregulated under low temperature, low nitrogen and low phosphorus conditions along the leaf gradient (tip – base). [file 1471-2164-14-442-S2.pdf]
